# Effective Rehabilitation of a Lisfranc Fracture in a 25-Year-Old Male Patient: A Case Report

**DOI:** 10.7759/cureus.60722

**Published:** 2024-05-20

**Authors:** Neha M Chitlange, Swapnil U Ramteke

**Affiliations:** 1 Department of Sports Physiotherapy, Ravi Nair Physiotherapy College, Datta Meghe Institute of Higher Education and Research, Wardha, IND

**Keywords:** case report, trauma, rehab protocol, physiotherapy, lisfranc fracture-dislocations

## Abstract

A dislocation or break of the tarsometatarsal joint in the foot is referred to as a Lisfranc fracture, sometimes called a Lisfranc injury. It can be caused by less stressful mechanisms like a twisting fall as well as high-energy events like car crashes or falls from heights. Swelling, bruises, and midfoot pain that gets worse when standing or walking are some of the symptoms. The damage may only affect the ligaments or the foot's bony structures. Nonoperative or surgical treatment may be part of the management, depending on how severe the injury is. In order to realign and stabilize the bones, open reduction internal fixation with Kirschner wires (K-wires) is a common surgical procedure. In this case, a 25-year-old male patient complained of left foot pain and wound. He gave a history of a left leg stuck in the harvester. Immediately, he was taken to a local hospital, where a dressing of his left foot was done. He was referred to a super specialty hospital where an investigation, like an X-ray, was done, which revealed a Lisfranc fracture. K-wire was applied to fix the Lisfranc fracture. Further on, rehabilitation was started to restore mobility, regain full range of motion, and develop muscle strength. American Orthopedic Foot and Ankle Score (AOFAS) and Lower Extremity Functional Scale (LEFS) were used as outcome measures.

## Introduction

French physician Jacques Lisfranc, who served in Napoleon's army in 1815, reported cases of secondary infection-related amputations via the tarsometatarsal joint [1]. A Lisfranc dislocation or injury usually refers to a range of injuries that affect the foot's tarsometatarsal joints [2]. The articulation between the cuneiform bones and the first to third metatarsal bones makes up the actual Lisfranc joint [3]. From complete tarsometatarsal displacement accompanied by fractures and ligamentous rips to partial sprains without displacement, joint injuries can range in severity [3]. Though Lisfranc injuries can occur in numerous parts of the foot, the isolated ligament that connects the medial cuneiform to the second metatarsal is the Lisfranc ligament [3]. Lisfranc joint injuries are uncommon. They are often misdiagnosed and mismanaged [1]. Early detection and prompt treatment are crucial because delaying diagnosis and care can result in midfoot arthritis, chronic pain, and functional instability [1]. Chronic disability and complications are still very likely, even in cases where they are identified early and treated appropriately [2].

Indirect and direct mechanisms are typically the two main reasons for this kind of injury. Crush injuries to the joint region resulting from events like auto accidents or workplace mishaps constitute a direct mechanism of injury [4]. Indirect injuries are more frequent than direct injuries and are frequently connected to involvement in sports [5]. In most cases, this mechanism of injury involves a longitudinal force while the foot is plantar flexed and rotates medially or laterally [5].

Lisfranc injuries are relatively uncommon. Although they make up 0.2% of all fractures, their prevalence is probably higher because they are often misdiagnosed [6]. The incidence of this injury is approximately one per 55,000 individuals per year [6]. Although it can happen at any age, the third decade of life is when it occurs most frequently. It is more common in males [6].

In order to achieve anatomic reduction, patients with displaced or unstable Lisfranc joint injuries must undergo surgery. An external fixator or Kirschner wires (K-wires) must be used for axial alignment and stabilization in cases where there is a significant displacement of the metatarsals [7]. K-wires are a better surgical option for managing Lisfranc fractures because they have been linked to a lower rate of complications than other fixation techniques [8]. K-wires can be removed rather easily without the need for a second surgical procedure, usually six to eight weeks following the initial surgery [8].

The clinical result of anatomical open reduction of Lisfranc fracture-dislocation depends critically on postoperative rehabilitation [9]. The rehabilitation strategy, which includes isometric calf muscle exercises to strengthen the dorsal extensors, focuses on regaining proprioception [9]. Clinically successful rehabilitation after foot injuries has focused on using exercises to restore muscle strength, joint range of motion, neuromuscular coordination, and gait mechanics [9].

## Case presentation

A 25-year-old male, farmer by occupation, came with a complaint of pain and a wound over his left foot for two days. He gave a history of his left leg getting stuck in the harvester in the evening. The pain was sharp, shooting, and continuous. It got aggravated on movements. The lacerated wound was present on the left foot, which was 8 × 5 × 4. He was taken to a local hospital where dressing was done. With the above complaint, he visited Acharya Vinoba Bhave Rural Hospital (AVBRH). An X-ray was done, diagnosed with a first metatarsal fracture with dislocation of the metatarsal navicular joint (International Classification of Diseases (ICD) Code S93. 324). He was advised to undergo surgery. K-wire fixation of the first and second metatarsal, wound debridement, and tendon repair on the left foot were performed. Post-operatively, the patient was treated with medications and was recommended for physiotherapy for rehabilitation, which aims to regain full mobility, develop muscle strength, and improve quality of life. The timeline is shown in Table 1.

**Table 1:** Timeline of events

Date	Events
21/11/23	The incident occurred at 8 pm.
22/11/23	Visited the hospital and got diagnosed with a Lisfranc fracture.
24/11/23	Got operated.
25/11/23	Physiotherapy started.

Clinical findings

**Figure 1:**
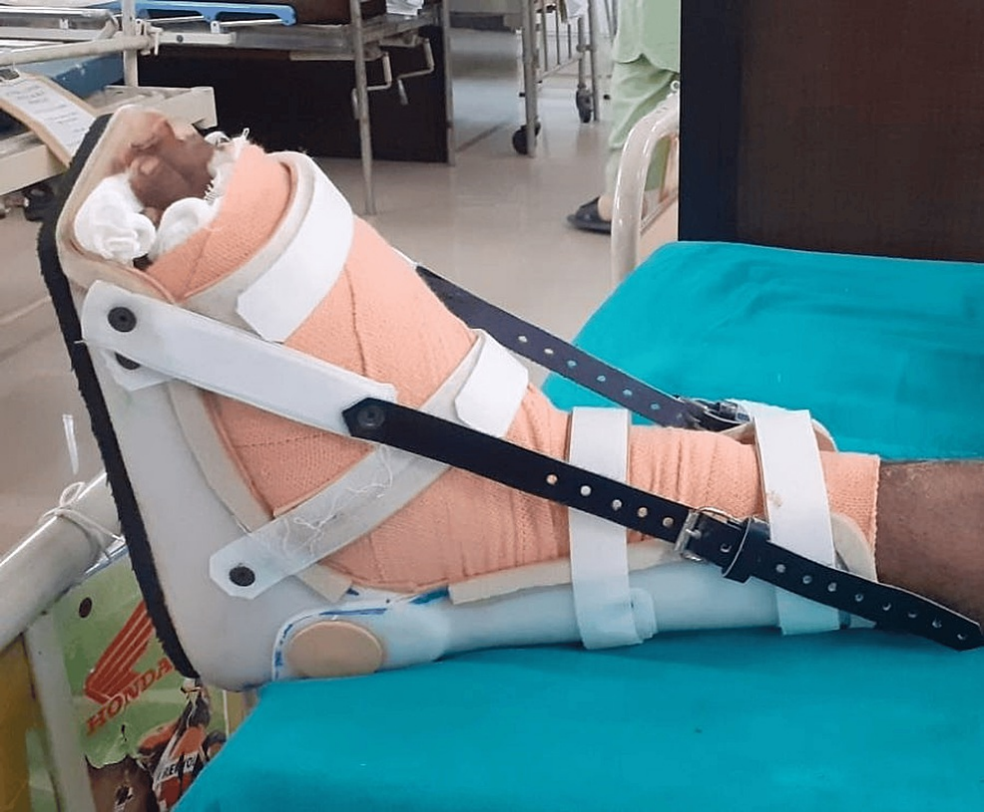
Ankle foot orthosis

The patient was seen lying supine and was conscious and well-oriented. Verbal consent was obtained from the patient before the physical examination. On inspection, it was observed that the left hip was slightly abducted, flexed, and externally rotated. The knee was somewhat flexed, and the ankle was in a neutral position. The left foot was bandaged from toes to mid-leg and was elevated on two pillows. Ankle foot orthosis was present, as seen in Figure 1. On palpation, grade 2 tenderness was marked over the anterior aspect of the foot, and severe pain was present at the operative site. The visual analog scale (VAS) score was 8/10 for activity and 5/10 for rest. The range of motion of the left hip and knee was normal. Manual muscle testing of the left hip and knee was three out of five. Range of motion and manual muscle testing of the ankle were done after the sixth week due to immobilization.

Medical management 

**Figure 2:**
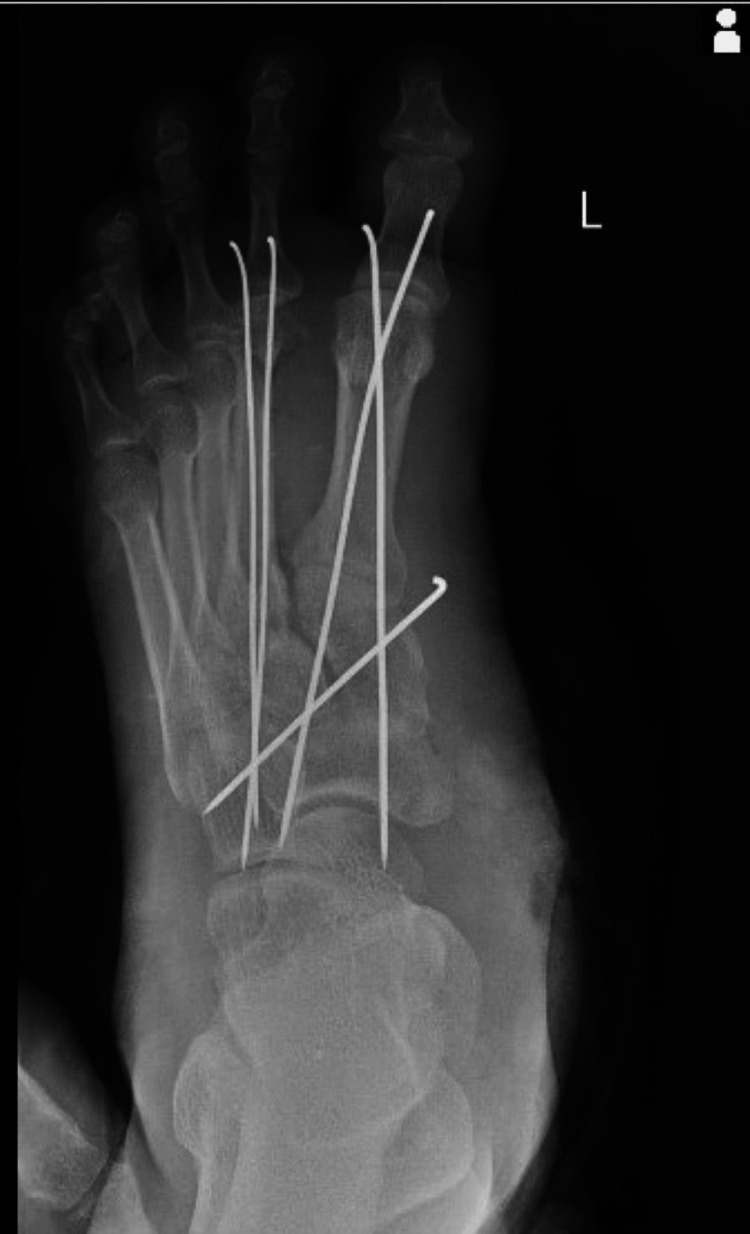
Post-operative X-ray with K-wire fixation

The patient visited AVBRH with major complaints of pain and wound for which he was admitted. An X-ray of the left ankle joint revealed a first metatarsal fracture with dislocation of the metatarsal navicular joint. K-wire fixation of the first and second metatarsal and wound debridement on left foot surgery were performed. After six weeks, the K-wire was removed. A postoperative X-ray is shown in Figure 2. After surgery, postoperative medication is shown in Table 2. The range of motion at baseline is offered in Table 3. The range of motion after three weeks of rehabilitation is shown in Table 4. Week-wise goals, physiotherapy management, and dosage are shown in Table 5. The outcome measure is shown in Table 6.

**Table 2:** Post-operative medications BD, two times a day; g, gram; Inj, Injection; IV, intravascular; mg, milligram; mL, milliliter; NS, normal saline; TDS, three times a day; Tab, tablet

Medications
Inj Ceftriaxone 1 g IV BD for three days
Tab Paracetamol 650 mg BD
Inj Amikacin 500 mg IV BD for three days
Inj Dynapar AQ 1 mL in 100 mL NS IV BD for three days
Tab Limcee 500 mg BD for 3 days
Tab Chymoral Forte 2 tabs TD for 3 days
Tab Trental 400 mg TD

**Table 3:** Range of motion of the left ankle

Joint	Movement	Week 6	Week 8
Ankle joint	Plantarflexion	0-15^0^	0-45^0^
	Dorsiflexion	0-5^0^	0-15^0^
	Inversion	0-10^0^	0-30^0^
	Eversion	0-5^0^	0-15^0^

**Table 4:** Manual muscle testing (according to Oxford grading) of left ankle 3-: muscle moves the joint against gravity but not through a full mechanical range of motion; 4+: muscle holds the joint against moderate to maximal resistance

Joint	Muscle	Week 6	Week 8
Ankle joint	Plantarflexor	3-	4+
	Dorsiflexor	3-	4+
	Invertors	3-	4+
	Evertors	3-	4+

**Table 5:** Physiotherapy protocol AROM, active range of motion; reps, repetition; sec, second; min, minutes; kg, kilogram

Phase	Physiotherapy goals	Therapeutic intervention	Dosage
Day 1 to week 1 (non-weight bearing)	To educate the patient	To guide the patient regarding the rehabilitation program and its effect. To make the patient aware of the preventive strategy during rehab.	Early ambulation, positioning, and resumption of activities of daily life education
	To maintain and improve ranges of lower limb	Straight leg raises exercise	Each exercise for 10 reps × 1 set (progress with 20 reps and 5-sec hold)
		Hip abduction exercise	
		Toe flexion-extension movements exercise	
		Dynamic quads	
	To maintain the strength of the lower limb	Static quads	10 reps × 1 set
		Static glutes	
		Static back	
	To promote relaxation	Jacobson relaxation	For 10 min
Week 2 (non-weight bearing)	To increase the strength of the lower limb	Straight leg raise	10 rep × 1 set with 5-sec hold (then progress with 10-sec hold)
		Dynamic quadriceps	
	To increase the strength of the upper limb	Shoulder and elbow (flexion, extension, abduction, adduction) with ½ kg weight cuff	10 reps × 1 set (progress with 1 then 2 kg weight cuff)
Weeks 4 to 6	To strengthen crutch muscle	Serratus anterior - straight arm plank	10 reps × 1 sets
	To initiate standing	Stand with walker	For 5-10 min
	To initiate walking	Walking with a walker with partial weight bearing on the ankle	Around bedside
Weeks 6 to 8 (K-wire was removed after six weeks.)	To reduce pain and swelling	Cryotherapy (ice pack)	For 15-20 min two times/day
		Neuromuscular electrical stimulator	Frequency: 30-100 Hz five days/week for 15 minutes
	To facilitate further healing of scar	Ultrasound	Intensity: 0.25-0.5 W/cm^2^ for 5 min
	To increase ankle range of motion	AROM of the ankle, i.e., plantarflexion/dorsiflexion, eversion/inversion	10 reps × 1 set
	To increase ankle strength	Isometrics of the ankle (plantarflexion/dorsiflexion, eversion/inversion)	10 reps × 1 set
	For ambulation	Walking with a walker with partial weight bearing on the ankle as tolerated	One round
Weeks 8 to 12	To increase ankle strength	Ankle (plantarflexion/dorsiflexion, eversion/inversion) with the band	Starting with yellow and progressing with red and green 10 reps × 1 set
	To increase balance and proprioception	Football rolling (under foot) in multi-direction	For 15 min (eyes open and progress with eyes closed)
		Single leg standing	10-15 sec with support (progress with unsupported)
	For gait training	Walking with a walker with full weight bearing on the ankle	One round (progress with or without a walker)
		Treadmill training	For 10 min

**Table 6:** Outcome measures

Scale	Week 6	Week 8
American Orthopaedic Foot and Ankle Score (AOFAS)	30/100	81/100
Lower Extremity Functional Scale (LEFS)	17/80	50/80

## Discussion

The displacement or dislocation of the metatarsal bones from the tarsus is a form of injury known as a Lisfranc injury [10]. It involves all three fractures, sprains, and dislocations simultaneously [10]. Trauma, either direct or indirect, may have caused the injury. When an external force acts on the foot, direct trauma is produced [11]. On the other hand, indirect trauma occurs when the foot twists after getting stuck in something [11]. Complicated situations arise from Lisfranc injury. The first through third metatarsals articulate the middle and lateral in the five tarsometatarsal joints that make up the Lisfranc joint [11]. Auto accidents and industrial accidents involving high energy can cause Lisfranc injuries, as can low-energy injuries sustained in sports [12]. On the top and bottom of the foot, there may be bruising, soreness, and swelling [13]. Among the available treatments are primary partial arthrodesis, midfoot fusion, and open reduction internal fixation (ORIF) [14]. When evaluated and treated effectively, patients with Lisfranc injuries can usually expect a positive outcome - a recovery to almost their pre-injury level [14].

After a Lisfranc fracture, strengthening the muscles around the foot and ankle is an important part of the rehabilitation process. Physical therapy plays a crucial role in managing pain, improving mobility, and restoring normal walking ability. Specific exercises can help in strengthening the muscles within the foot. Additionally, manual therapy and neuromuscular electrical stimulation may be used to facilitate muscle strengthening and improve mobility [15]. Proprioception and balance training can be beneficial in the rehabilitation of midfoot fractures. It is observed that balance training activities, like single-limb standing, are successful when one progresses them by adjusting arm position, closing eyes, and adding an unstable surface [16]. A case report showed that a patient with a Jones fracture was treated with continuous cryotherapy and proprioceptive training, and the patient was able to walk normally with full weight bearing after 12 days of treatment [17]. Low-intensity pulsed ultrasound has been found to be effective in enhancing the healing rate of scars, including those in the foot and ankle [18]. Muscle strengthening exercises are crucial in rehab regimens, enhancing flexibility, stability, and confidence while also preventing falls and restoring mobility, thereby improving patients' quality of life and reducing discomfort [19].

## Conclusions

The rehabilitation protocol that was employed produced favorable results. It can be a useful guide for cases that are similar to this one. This report emphasizes how crucial customized rehabilitation plans are to helping patients with Lisfranc fractures recover favorably. In the management of such injuries, it also emphasizes the value of interdisciplinary approaches as well as patient-specific care. This study demonstrates the beneficial effects of early physiotherapy therapy in conjunction with definitive surgical therapies on fracture patients' clinical outcomes.
